# Diabetes surveillance – Laying the groundwork for non-communicable disease surveillance in Germany

**DOI:** 10.25646/12199

**Published:** 2024-06-26

**Authors:** Christa Scheidt-Nave, Christin Heidemann, Lukas Reitzle, Maike Buchmann, Thomas Ziese, Andrea Icks

**Affiliations:** 1 Robert Koch Institute, Department of Epidemiology and Health Monitoring, Berlin, Germany; 2 Institute for Health Services Research and Health Economics, German Diabetes Centre, Leibniz Institute for Diabetes Research, Düsseldorf, Germany

Diabetes mellitus is a chronic disorder of blood sugar metabolism that requires lifestyle adjustments and often lifelong treatment with blood sugar-lowering medications. Even though treatment options and care services are constantly being improved, diabetes increases the risk of complications reducing quality of life and life expectancy. The incidence of type 2 diabetes mellitus (T2DM) in particular has risen sharply worldwide in recent decades. T2DM shares important known and potentially modifiable risk factors with other common non-communicable diseases (NCDs). These include individual risk factors such as obesity, lack of exercise, smoking and an unhealthy diet, but also social determinants such as education, living and working conditions. National and international strategies for the prevention of T2DM and other NCDs therefore require cross-disease approaches and a population-representative, scientifically sound data and information basis that is comparable over time in order to support the planning and scientific monitoring of health policy measures [1].

In order to ensure a continuous and systematic assessment of the epidemiological situation, the burden of disease and the need for prevention and care in connection with diabetes in Germany, the Federal Ministry of Health in Germany (BMG) has been funding the establishment of diabetes surveillance at the Robert Koch Institute (RKI) and its expansion into NCD surveillance since 2015 [2]. Key features of the surveillance system are a scientific framework with clearly defined fields of action and core health measures (indicators) as well as a population-based database, which is being successively expanded. Primary data from nationwide studies conducted by the RKI as well as secondary data from official statistics, disease registries and statutory health insurance (SHI) are taken into account. Project results are disseminated via the diabetes surveillance website as well as topic-specific reports and scientific publications. Parallel to diabetes surveillance, the BMG funded the development of the national education and communication strategy on diabetes mellitus by the Federal Centre for Health Education (BZgA). To implement this strategy, a nationwide cooperation network “Diabetesnetz Deutschland - gemeinsam gesünder” is currently being set up, in which the RKI is involved with the experience and results from diabetes surveillance. By the end of 2024, diabetes surveillance will be expanded in a first step to include further indicators for NCD surveillance. For mental health indicators, this will be done in cooperation between diabetes surveillance and a project funded separately by the BMG from 2019 to 2023 to establish mental health surveillance in Germany. The articles published in the Journal of Health Monitoring in the second quarter of 2024 [3, 4, 5, 6] illustrate the relevance and potential of indicator-based NCD surveillance as a basis for coordinated and evidence-based prevention and care of diabetes mellitus and other important NCDs.

In their article on the prevalence of gestational diabetes mellitus (GDM) in Germany, Reitzle et al. [3] continue analyses of temporal trends in a core indicator of diabetes surveillance. For the first time, regional socioeconomic differences are also analysed. GDM is a generally transient disorder of maternal blood glucose metabolism during pregnancy, which increases the risk of birth complications and future maternal risk of T2DM. According to recent findings, GDM may also increase the risk of childhood obesity. For the analyses, data from the external inpatient quality assurance for obstetrics (perinatal medicine) 2013–2021 were linked to the German Index of Socioeconomic Deprivation (GISD) at regional level using the first four digits of the zip code. Since the introduction of two-stage screening for GDM in Germany in 2012, there has been a continuous increase in the prevalence of GDM, regardless of the mother’s age. For 2021, the prevalence is estimated at 8.5 %, which corresponds to more than 63,000 women with GDM. As the estimates are influenced by the completeness of the documentation of GDM in the maternity record, it can be assumed that the prevalence is still underestimated. In regions with high socioeconomic deprivation, significantly higher prevalence rates can be observed than in regions with low deprivation, whereby health inequalities in GDM prevalence have increased over time. Whether this is due to catchup effects in GDM screening among socially disadvantaged women or also to increasing social differences in the prevalence of important GDM risk factors such as pre-conceptional maternal obesity or excessive weight gain during pregnancy cannot be clarified with the available data. The results are consistent with an increase in the prevalence of GDM observed worldwide. They underline the need for life-stage specific approaches to promote cardiometabolic health and reduce health inequalities. For targeted prevention of GDM and subsequent complications, information on individual maternal risk factors for GDM and the screening rate must be made continuously available for NCD surveillance via perinatal statistics in addition to information on GDM prevalence and social context factors.

In their article, Heidemann et al. [4] provide a comprehensive insight into the health situation and healthcare of adults 45 years of age and older with T2DM during the COVID-19 pandemic. Analyses are based on data collected among adults with diabetes mellitus in the population-based telephone survey GEDA 2021/2022-Diabetes, which was conducted at the RKI for diabetes surveillance at the end of the second year of the pandemic (December 2021 to April 2022). In addition to current information on numerous diabetes surveillance core indicators, information was also collected on some mental health surveillance indicators. Around 50 % of adults with T2DM rate their general health as very good or good. Almost a third of adults with T2DM who reported SARS-CoV-2 infection rate their general health as worse than before the pandemic, compared to just under a quarter without infection. Comparable data from surveys of the general population show that a deterioration in health is reported significantly less frequently among people with SARS-CoV-2 infection, whereas there are no differences among people without infection. These results appear plausible in view of the known interactions between COVID-19 and pre-existing cardiometabolic diseases. Compared to the general population, a significantly lower proportion of adults with T2DM rate their mental health as very good or excellent, whereas there were no significant differences in the presence of anxiety disorders, perceived social support or feelings of loneliness. Around 17 % of women and men with T2DM have depressive symptoms. However, there is a lack of comparative data on the general population for the same period. Assessments of general and mental health in adults with T2DM are consistently worse in association with lower education. The results from GEDA 2021/2022-Diabetes on selected indicators of self-management and quality of care in adults with T2DM can be compared with results from previous health surveys from 2008–2011 and 2014/2015 and are almost unchanged. While there are apparently no improvements over time, identifying pandemic-related changes would have required monitoring at short time intervals. Among adults with T2DM surveyed, only two thirds report to have participated in diabetes education programmes or to adhere to recommended self-controls of blood sugar and feet as well as medical check-ups of the eyes and feet. The indicator for self-assessed quality of care assesses various aspects of patient-centred care using an instrument validated in Germany (Patient Assessment of Chronic Illness Care, PACIC). Results of a survey of adults with diabetes mellitus from 2017 show that even before the pandemic, the self-assessed quality of care was only rated as mediocre on average. In GEDA 2021/2022-Diabetes, these results are confirmed and a slight deterioration is observed in men but not in women. Consistently, almost no differences by education were observed for the care indicators, which is in line with an earlier analysis [7].

The fact sheet by Tuncer et al. [5] analyses temporal trends and socio-spatial differences in diabetes-related lower limb amputation rates in relation to the total population. This indicator of diabetes surveillance records one of the most serious diabetes-related long-term complications and is internationally established as an indicator for the quality of care. It measures the annual rate of hospital admissions due to diabetes-related lower limb amputations per 100,000 inhabitants. A distinction is made between minor and major amputations, i.e. amputations below or above the ankle region. The analyses are based on national diagnosis-related groups (DRG) hospital statistics of the Federal Statistical Office for the years 2015–2022, which were linked to the GISD at district level. Among women, there is a decrease in both the annual diabetes-related major and minor amputation rates in the period under review. However, among men major amputation rates slightly increased following a decrease between 2015 and 2020 and minor amputation rates significantly increased after fluctuations between 2015 and 2020. In accordance with results from analyses of SHI data, this shows possible effects of the pandemic on the quality of diabetes care. There are pronounced differences according to sex and social deprivation, with significantly higher amputation rates among men compared to women and in regions with high compared to regions with low social deprivation. The extent to which these results reflect sex differences and health inequalities in diabetes prevalence could not be analysed in this study. There is no doubt that the regional level is crucial for the planning, implementation and scientific monitoring of prevention programs. As part of NCD surveillance, regionalized analyses need to be further developed considering primary and secondary prevention in context and taking individual as well as contextual factors into account.

Buchmann et al. [6] investigate associations between several diabetes surveillance core indicators and selected migration-related characteristics in adults living in Germany with Croatian, Italian, Polish, Syrian or Turkish citizenship. The data were collected in the context of the multilingual, multimodal RKI survey GEDA Fokus (2021/2022). For the analyses, migration-related variables that are known to be associated with health inequalities were considered: self-reported German language proficiency, experiences of discrimination in everyday life or in the health and care sector, and a sense of belonging to the society in Germany. Regardless of sociodemographic factors (age, sex, education) and citizenship as recorded in residents’ registries, lower self-assessed German language proficiency was associated with a higher 5-year risk of T2DM as estimated by the German Diabetes Risk Score. Among respondents with T2DM between 45 and 79 years of age, diabetes-related organ complications and depressive symptoms were observed significantly more frequently among those with more frequent experiences of discrimination in the health and care sector. For diabetes-specific care indicators (blood glucose monitoring, drug therapy) and the frequency of cardiovascular comorbidities, no associations were observed with the migration-related characteristics considered. The results demonstrate that health inequalities in connection with the migration-related characteristics considered are also evident in relation to the primary and secondary prevention of diabetes. Surveillance of diabetes and other NCDs should include the entire population, i.e. also people with a history of migration. In order to support the development of services tailored to the needs of people with a history of migration, future health surveys need to collect information in addition to the migration-related determinants considered, e.g. information on health knowledge and information needs and specific living conditions.

Diabetes surveillance has laid important foundations for NCD surveillance. Links between T2DM and other major physical and mental NCDs were taken into account, as were the importance of social determinants and the need for life-stage specific and regional approaches to prevention [1, 2, 7]. Concepts and indicators for modifiable individual and contextual risk factors were discussed and consented based on scientific exchange and networking at the national and international level [2, 8]. Together with the results of diabetes surveillance to date, the results of NCD surveillance will be incorporated into a comprehensive indicator-based health information system in Germany. This will provide an indispensable data and information basis for the planning and scientific monitoring of prevention measures as well as for health and risk communication. For health promotion and NCD prevention in Germany, it will be crucial to use, further develop and consolidate NCD surveillance in the context of a concerted national strategy to combat NCDs in Germany. This requires a clear focus on health goals and target achievement criteria, a continuous dialogue at the regional, national and international level, and the further enhancement of data sources for NCD surveillance, including population-based health interview and examination surveys [9].
